# Topological signatures in the entanglement of a topological insulator-quantum dot hybrid

**DOI:** 10.1038/s41598-022-24939-3

**Published:** 2022-12-02

**Authors:** L. A. Castro-Enríquez, A. Martín-Ruiz, Mauro Cambiaso

**Affiliations:** 1 Departamento de Ciencias Físicas, Universidad Andres Bello, Av. Sazié 2212, 8370136 Santiago, Chile; 2 Instituto de Ciencias Nucleares, Universidad Nacional Autónoma de México, 04510 Ciudad de México, México

**Keywords:** Nanoscale materials, Quantum physics, Quantum optics

## Abstract

In the present work, we consider a hybrid plexciton composed of a semiconductor quantum dot interacting with a topological insulator nanoparticle subject to an external magnetic field. Due to the topological magnetoelectricity of the nanoparticle, long-living plasmonic surface modes are induced, which are quantized and coupled with the quantum dot through its polarization operator. We consider the hybrid as an open quantum system, such that environment effects are accounted by the master equation in the Born–Markov approximation. Then, we apply the Peres’ positive partial transpose criterion to quantify the entanglement of the hybrid. We show that this entanglement is a direct signature of the $\mathbb {Z}_2$ invariant of topological insulators.

## Introduction

Topological insulators (TIs) has been one of the most active research areas in the last decade both from the theoretical and experimental sides.The study of TIs has elucidated further subtleties in the electronic band structure of solids, deepening our understanding of the phases of quantum matter, leading to far-reaching new opportunities. TIs are characterized by a gapped bulk and time-reversal symmetry protected boundary modes that are robust against disorder^1^, which have found applications in spintronics^2^, electrical memory devices^3,4^ and advanced magnetoelectronic and optoelectronic devices as well^5,6^. Apart from their characteristic surface modes, TIs also exhibits an unusual electromagnetic response, which is described by a topological field theory akin to axion electrodynamics^7^.

The notion of topological order allows to distinguish phases of matter beyond the Landau paradigm of local order parameters associated with spontaneous symmetry breaking. Macroscopically, topological order is defined by robust ground state degeneracy and quantized Berry phases of degenerate ground states^1^. In the case of TIs the topological invariant enters into the effective topological field theory as the axion coupling, and hence their response to applied electromagnetic fields evinces the nontrivial topological order of the material. Microscopically, topological order corresponds to patterns of long-range quantum entanglement: states with different topological orders or different patterns of long-range entanglements cannot change from one to another without a phase transition. Indeed, regardless of whether the quantum phases are differentiated by local order parameters or by topological order, the key point is that to go from one phase to another, the system must undergo a phase transition. As is well known, the behaviour of several thermodynamic quantities are highly sensitive to phase transitions. On the other hand, as topological order is related with long-range correlations between the constituents of the system, it is no wonder that entanglement might reveal a signature of the quantum phase transition between different topological orders. In fact, recently, the entanglement entropy has been developed as a way to characterize topological phases based on certain bulk properties of just the ground-state wave function, specifically, the properties of its entanglement spectrum^8^. For example, the 3D TI Bi$_2$Te$_3$ can host different structures of entanglement in the bulk and the surface: the surface states lives as maximally entangled states, while the bulk states do not^9^.

**Figure 1:**
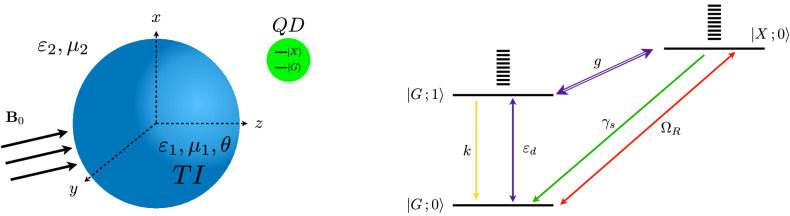
(Left) Schematics of the hybrid plexciton composed of a topological insulator nanoparticle and a semiconductor quantum dot in the presence of an external magnetic field. (Right) TI-QD tower of states truncated for $m=0,1$, the states are connected by the amplitude $\varepsilon _{d}$ of the TI electric field, the total decay rate *k* of the TI, the coupling strength parameter *g*, the spontaneous emission rate $\gamma _{s}$ of the QD and the Rabi frequency $\Omega _{R}$.

Recent advances in nanostructures such as semiconductor quantum dots (SQDs) and metal nanoparticles has proven to be of fundamental importance in modern nanoscience and nanotechnology for their applications in photonics and optoelectronics. In particular, hybrid systems where SQDs couple to plasmonic nanostructures have attracted great attention mainly because it provides an intuitive picture for highly sensitive detectors^10–12^ and eventually leads to innovative new devices, such as DNA sensors^13^, laser systems without cavities^14^ and manipulation of heat generation in metal nanoparticles (MNPs)^15^. In recent years, there has been an ongoing effort to generate entanglement between quantum dots coupled to plasmonic nanostructures in a single cavity^16,17^, as well as its experimental detection^18^. In this paper, we suggest that these advances, together with the recent progress in the fabrication of nanostructured devices made from TI materials (i.e. TI nanoparticles^19–22^ and nanowires^23–27^), can be used to form a topological hybrid plexciton, whose entanglement pattern can serve as a direct probe to detect the bulk topology of 3D TIs. Along this vein, Fano resonances has been predicted to be a measure of the $\mathbb {Z}_2$ invariant of TIs by using a similar topological hybrid plexciton^28,29^. Clearly, the entanglement we refer to is indeed different from the entanglement spectrum proposed to characterize the topological order. The former is related with the quantification of exciton-photon entanglement. In this work we consider an hybrid plexciton composed of a SQD interacting with a TI nanoparticle subject to an applied magnetic field (Fig. 1). The induced long-living plasmonic surface modes are quantized and then coupled with the quantum dot through its polarization operator. We also account for the damping and environment effects. Using the Lindblad’s master equation in the Born–Markov approximation, we apply the Peres positive partial transpose criterion to quantify the entanglement of the hybrid. Our theoretical results show that the entanglement is sensitive to the topological magnetoelectric polarization for both, the longitudinal and the transverse couplings.

## Results

### Electrodynamics of topological insulators: electromagnetic response and quantization

The electromagnetic response of a 3D topological insulator is described by two terms. The first is the usual Maxwell Lagrangian $\mathscr {L} _{M} = \tfrac{1}{2} \left( \varepsilon \varepsilon _{0} \mathbf{{E}} ^{2} - \mathbf{{B}}^{2} / \mu \mu _{0} \right)$ from which Maxwell equations can be derived, and the second is the axionic $\theta$-term (in SI units) $\mathscr {L} _{\theta } = ( \alpha / \pi Z _{0} ) \theta \, \mathbf{{E}} \cdot \mathbf{{B}}$, where $\alpha \simeq 1 / 137$ is the fine structure constant, $Z _{0} = \sqrt{\mu _{0} / \varepsilon _{0}}$ is the impedance of free space and $\theta$ is the topological magnetoelectric polarizability (TMEP), whose origin is quantum-mechanical. In the presence of TR symmetry, $\theta$ takes a quantized value $\pi$ (mod $2 \pi$) for TIs and $\theta = 0$ in trivial insulators. A number of magnetoelectric effects have been predicted on the basis of this theory^30–38^. However, it has been experimentally verified only through the measurement of Kerr and Faraday angles at the surface of a strained HgTe 3D TI^39^.

In the problem at hand, with the aim of quantizing the electromagnetic field modes, we begin with the classical electromagnetic description of a spherical 3D TI embedded in a medium with dielectric constant $\varepsilon _{2}$ and subjected to an externally applied monochromatic magnetic field. We consider the TI characterized by a dielectric function $\varepsilon _{1}(\omega )$, the TMEP $\theta$, and the magnetic permeability $\mu \simeq 1$. Assuming that the wavelength of the applied field is large as compared with the TI size, we can work in the time harmonic approximation, for which the electromagnetic fields can be written as $\mathbf{{E}} (\mathbf{{r}} , t) = \text{ Re } \big \{\ \!\! \mathbf{{E}} (\mathbf{{r}}) e ^{- i \omega t} \big \}\ \!\!$ and $\mathbf{{B}} (\mathbf{{r}} , t) \! = \! \text{ Re } \big \{\ \!\! \mathbf{{B}} (\mathbf{{r}}) e ^{- i \omega t} \big \}\ \!\!$, where $\mathbf{{E}} (\mathbf{{r}})$ and $\mathbf{{B}} (\mathbf{{r}})$ are the fields associated with the static solutions to the equations of axion electrodynamics. Due to the TME effect, the magnetic field polarises the TI, which can be described by the electric field

1 $$\begin{aligned} \mathbf{{E}} (\mathbf{{r}} , \omega ) = - \frac{\tilde{\alpha }c}{2 \varepsilon _{2} + \varepsilon _{1}(\omega ) + (2/3) \tilde{\alpha } ^{2}} \,\sum _{i} B_{0i} \, \mathbf{{G}} _{i} (\mathbf{{r}}) , \quad \mathbf{{G}} _{i} (\mathbf{{r}}) = \left\{ \begin{array}{c} \hat{\mathbf{{e}}} _{i} \\ - \frac{R ^{3}}{r ^{3}} \left[ 3\,(\hat{\mathbf{{e}}} _{r} \cdot \hat{\mathbf{{e}}} _{i})\, \hat{\mathbf{{e}}} _{r} - \hat{\mathbf{{e}}} _{i} \right] \end{array} \right. \begin{array}{c} r<R \\ r>R \end{array} , \end{aligned}$$

where $\tilde{\alpha } = \alpha \cdot (\theta / \pi ) = (2l+1)/137$ with $l=0,1,2,\ldots$, *c* is the speed of light and $B_{0i}$ is the *i*-th component of the external magnetic field. We observe that the electric field inside the TI is uniform as that of a uniformly polarised sphere, whilst the electric field outside is dipolar. Certainly, this electric field is a direct manifestation of the nontrivial topological order of the TI. On the other hand, the magnetic field can be expressed as the externally applied field $\mathbf{{B}} _{0}$ plus a dipolar field which is proportional to the electric field (1), i.e. $\mathbf{{B}} (\mathbf{{r}} , \omega ) = - (\tilde{\alpha } / 3c) \xi (r) \, \mathbf{{E}} (\mathbf{{r}} , \omega )$, where $\xi (r) = 1$ for $r>R$ and $\xi (r) = -2$ for $r<R$. We note that these fields satisfy the orthogonality $\int \mathbf{{F}} _{i} \cdot \mathbf{{F}} _{j} \, d ^{3} \mathbf{{r}} = 0$ for $i \ne j$, where $\mathbf{{F}}$ is either $\mathbf{{E}}$ or $\mathbf{{B}}$.

The time-dependent fields can be obtained by Fourier transforming the above results. To do this, we require a model for the dielectric function $\varepsilon _{1}(\omega )$. Because of the low concentration of free carriers in insulators, the dielectric function $\varepsilon _{1}(\omega )$ can be well modeled by $\varepsilon _{1}(\omega ) - 1 = \omega _{e} ^{2} [ \omega _{R} ^{2} - \omega (\omega + i \gamma _{0}) ] ^{-1}$, where $\omega _{R}$ and $\omega _{e}$ are the resonant and natural frequencies, respectively. The damping parameter $\gamma _{0}$ (for which $\gamma _{0} \ll \omega _{R}$) accounts for energy dissipation due to Ohmic losses in the TI. When $\gamma _{0} \ll \omega$ the electric field (1) follows a Lorentzian spectrum:

2 $$\begin{aligned} \mathbf{{E}} (\mathbf{{r}} , \omega ) = - \eta \frac{\omega _{0} ^{2}/2 \Omega }{\omega - \Omega + i \gamma _{0}/2} \sum _{i} c B_{0i} \, \mathbf{{G}} _{i} (\mathbf{{r}}) , \quad \eta = \frac{\tilde{\alpha }}{2\varepsilon _{2} + 1 + (2/3) \tilde{\alpha } ^{2}} , \end{aligned}$$

where $\omega _{0} = \omega _{e} \sqrt{\eta / \tilde{\alpha }}$ and $\Omega ^{2} = \omega _{R} ^{2} + \omega _{0} ^{2}$. The Lorentzian approximation to the TI spectral response is appropriate when it interacts with a dipole whose resonant frequency is close to plasmon resonance^40^, which is the regime of interest for this article.

### Quantum optical model for TI-dipole interaction

The electromagnetic fields from the previous section are not localised in space. The electric field arises as a TME response of the TI to the external magnetic field carrying a monochromatic component $\textbf{B}_0(\textbf{r},t) = \text{ Re } \big \{ \textbf{B}_0 e ^{- i \omega t} \big \}$, comprising a wave that extends infinitely in space. As we are concerned with a quantum mechanical description of the TI’s response, we need to quantise the arising EM fields due to the TME polarisation of the TI. However, for quantizing the field modes we require localised solutions. These can be obtained by exciting the TI with an impulse function, since after the impulse has ended, only the localised modes will remain. If the TI is excited by an impulse input of the form $B _{0} (t) = B _{0} \delta (t)$ the time-domain electric field of the TI is

3 $$\begin{aligned} \mathbf{{E}} (\mathbf{{r}} , t ) = \sum _{i=x,y,z} \Lambda _{i} \mathbf{{G}} _{i} (\mathbf{{r}}) \, \sin (\Omega t) \, e ^{- \gamma _{0} t / 2 } , \quad \Lambda _{i} = -\eta \, (\omega _{0} ^{2} / 2 \Omega )\, B _{0i}\,c . \end{aligned}$$

By the way, the same proportionality between the electric and magnetic fields still holds in the time domain. To model the TI-dipole interaction quantum mechanically we will consider a full open system consisting of a closed sub-system formed by the TI-QD hybrid (plus their mutual interaction) interacting with an environment. We will describe each component below, but at this point we argue that the Ohmic dissipation is lost from the closed sub-system into the environment/bath creating phonon excitations from the bath’s vacuum. Thus, for the purpose of quantizing the EM fields, it proves useful to omit the damping parameter $\gamma _{0}$ in Eq. (3) which accounts for energy dissipation due to Ohmic losses present in matter, provided we include it afterwards as described above. In this context, dampings can be treated using Lindblad’s master equation formalism and we will do so in the next section.

Having omitted $\gamma _{0}$ in Eq. (3), the confined EM field modes on the TI’s surface will be steady-state sinusoidal functions. Noting that from Eq. (3) the Cartesian components of the confined surface fields modes are orthogonal, we may quantize each component individually. The quantization programme is along the lines of canonical quantization, with, however, a slight modification. We consider initially the energy of the *i*-th mode of the EM fields in a dispersive media: $U _{i} = \frac{\varepsilon _{0}}{2} \int \left[ \textbf{E} ^{2} _{i}\, \frac{d( \omega \varepsilon )}{d \omega } + \frac{1}{\mu }\,\textbf{B} ^{2} _{i} \right] \, d ^{3} \textbf{r}$^41^, and from here read a classical Hamiltonian in which the electric and magnetic fields play the role of canonically conjugated variables that, after promotion to quantum operators with defined commutation relations, yields the Hamiltonian of a quantum mechanical harmonic oscillator. However, such procedure is untenable. The magnetic field is reduced by a factor of $\alpha ^{2} \sim 10^{-5}$ in comparison with the electric field, thus the energy of the *i*-th mode is given by

4 $$\begin{aligned}U _{i}&= \frac{\varepsilon _{0}}{2} \Lambda _{i} ^{2} \sin ^{2} ( \Omega \,t) \int \vert \textbf{G} _{i} (\textbf{r}) \vert ^{2} \, \frac{d\text{ Re }( \omega \varepsilon )}{d \omega } \bigg | _{\omega = \Omega } \, d ^{3} \textbf{r} \, \equiv \,\hbar \Omega A _{i} ^{2} \sin ^{2} ( \Omega \,t), \end{aligned}$$

where we have introduced the normalized amplitude $A _{i} = \Lambda _{i} / N$, with $N^{-2} = \frac{\varepsilon _{0}}{2 \hbar \Omega } \int \, d ^{3} \textbf{r}\, \vert \textbf{G} _{i} (\textbf{r}) \vert ^{2} \, \frac{d{{\textrm{Re}}}( \omega \varepsilon )}{d \omega } \Big | _{\omega = \Omega }$. But, this will not lend for an appropriate interpretation as the Hamiltonian of quantum harmonic oscillator with the electric and magnetic fields are the canonical variables, because the magnetic contribution will be missing, i.e., as the electric potential energy of the *i*-th mode is not constant in time it cannot account for the total energy of the system, that must be conserved. The energetic balance tells us that in order for the total energy to be conserved, the electric potential energy $U_i$ must be continually transforming to some other form, say $K_i$, and vice-versa. From Eq. (4), we identify $K_i=\hbar \Omega A _{i} ^{2} \cos ^{2} ( \Omega \,t)$, which we can physically understand as a kinetic energy associated with the currents flowing in the TI’s surface driven by the *i*-th mode. Having done so, we can indeed, carry out a canonical quantization programme by considering a time-dependent field amplitude $\mathscr {A} _{i} (t) = A _{i} \sin (\Omega \,t)$. Thus the Hamiltonian of the confined field modes is given by $H _{i} = (\hbar /\Omega )\,\dot{\mathscr {A}} _{i} ^{2} + \Omega ^{2} \mathscr {A} _{i} ^{2}$. The two variables $\mathscr {A}_{i}$ and $\dot{\mathscr {A}}_{i}$ form a pair of canonical conjugate variables that can be promoted to quantum operators as $\mathscr {A}_{i}\rightarrow \hat{x}_{i}$ and $2\hbar \dot{\mathscr {A}}_{i}/\Omega \rightarrow \hat{p} _{i}$, that satisfy the commutation relations $\left[ \hat{x}_{i},\,\hat{p}_{j}\right] =i\delta _{ij}\hbar$. Introducing the creation and annihilation bosonic operators ${\hat{a}} _{i} = {\hat{x}} _{i} + (i/2 \hbar ) {\hat{p}} _{i}\,\,\, \text{and} \,\,\,{\hat{a}} _{i} ^{\dagger } = {\hat{x}} _{i} - (i/2 \hbar ) {\hat{p}} _{i}$^42^, and using the fact that the modes are orthogonal, we obtain the quantum Hamiltonian that describes the TI surface modes

5 $$\begin{aligned} \hat{H}_{\textrm{TI}} = \hbar \, \Omega \! \sum _{i = x,y,z} \left( \hat{a} ^{\dagger } _{i} \hat{a} _{i} + 1/2 \right). \end{aligned}$$

The above treatment also leads to an expression for the quantized electric field. In fact, one can show that $\Lambda _{i} = (N/2)\,(\hat{a}_{i}+\hat{a}_{i}^{\dag })/\sin (\Omega \,t)$. Therefore, the quantized electric field in the steady-state condition, Eq. (3), reads

6 $$\begin{aligned} \varvec{\hat{E}}_{\textrm{TI}}(\textbf{r},t) = \sqrt{\frac{\hbar \Omega }{2\varepsilon _{0}V_{m}}} \sum _{i=x,y,z}(\hat{a}_{i}+\hat{a}_{i}^{\dag })\, \textbf{Y}_{i}(\textbf{r}),\,\,\, V_{m} \approx \frac{4\pi R^{3}}{3}\, \frac{4\,\varepsilon _{2} + 2}{2\,\varepsilon _{2} + 2}, \end{aligned}$$

where $$\textbf{Y}_{i}(\textbf{r}) = \textbf{G}_{i}(\textbf{r})/\mathscr {U}_{0}$$ is a re-scaled function and $$V_{m}$$ is the mode-volume defined as the ratio between the total energy to the energy density inside the TI, i. e., $$V_{m} = [\int \,d ^{3} \textbf{r}\,\vert \textbf{G} _{i} (\textbf{r}) \vert ^{2} \, d\,\text{ Re }( \omega \varepsilon )/d \omega \vert _{\omega = \Omega }]\,/\, \mathscr {U} _{0}$$, with $$\mathscr {U} _{0} = \vert \textbf{G}_{i}(0)\vert ^{2}\, d\,\text{ Re }(\omega \varepsilon _{1})/ d\omega \vert _{\omega =\Omega }$$. It is worth to mention that the quantized electric field, Eq. (6), depends on the electric field per photon $$\text{ E}_{0} = \sqrt{\hbar \Omega /2\varepsilon _{0}V_{m}}$$, but not on the strength of the impulse magnetic field. Having obtained the quantized electric field, we proceed to deduce the Hamiltonian of TI-QD interaction. When the distance between the TI and the QD is larger than the radius of the TI, the dipolar approximation provides a reasonable result for the interaction strength given by $$\hat{H}_{\textrm{int}} = - \hat{\textbf{p}}\cdot \hat{\textbf{E}}_{\textrm{TI}}$$^28,29^, with $$\hat{\textbf{p}}$$ the dipole operator and $$\hat{\textbf{E}}_{\textrm{TI}}$$ the quantized electric field in Eq. (6). In this paper, we consider a spherically symmetric QD, therefore, the matrix elements of the dipole operator, $$\hat{\textbf{p}}_{\textrm{nm}} = \langle n|\hat{\textbf{p}}|m\rangle $$, point along the *x*, *y* or *z* axis. In this way, $$\hat{H}_{\textrm{int}}$$ couples field and dipole operators pointing along the same direction, i. e., the dipole is excited only in one specific direction and couples to one of the TI bosonic surface modes. Applying the two-level approximation, the dipole operator can be written as $$\hat{\textbf{p}} = d (\hat{\sigma }^{+} + \hat{\sigma 
}^{-})\hat{\textbf{e}}_{i=x,y,z}$$, where *d* is the dipole moment of the transition and $$\hat{\sigma }^{+}$$ ($$\hat{\sigma }^{-}$$) is the Pauli raising (lowering) operator. In brief, we treat the dipole and the TI as a two level-system interacting with a single bosonic mode whose field points in the longitudinal or transverse direction. Thus, the interaction Hamiltonian takes the form

7 $$\begin{aligned} \hat{H}_{\textrm{int}} = \hbar g(r) (\hat{\sigma }^{+}\hat{a} + \hat{\sigma }^{-}\hat{a}^{\dag }), \end{aligned}$$

where we applied the rotating wave approximation leaving only the energy-conservative terms. It is worth to mention that the coupling strength *g*(*r*) depends on whether the TI’s (electric) field points parallel to the dipole (which we call longitudinal coupling, LC), or transverse to it (transverse coupling, TC). Explicitly, the coupling strength reads

8 $$\begin{aligned} g(r) = \left\{ \begin{array}{l} +2 \, \frac{d}{\hbar } \sqrt{\frac{\hbar \Omega }{2 \varepsilon _{0} V _{m} \mathscr {U} _{0}}} \frac{R ^{3}}{r ^{3}} \\ -1 \, \frac{d}{\hbar } \sqrt{\frac{\hbar \Omega }{2 \varepsilon _{0} V _{m} \mathscr {U} _{0}}} \frac{R ^{3}}{r ^{3}} \end{array} \right. \begin{array}{l} \text{ LC }, \\ \text{ TC }. \end{array} \end{aligned}$$

We observe that as the LC is twice as strong as the TC, it should be a better candidate for experimental detection than TC. All in all the Hamiltonian describing the TI-QD closed system is $\hat{H} = \hat{H}_{\textrm{TI}} + \hat{H}_{\textrm{dip}} + \hat{H}_{\textrm{int}}$, where $\hat{H}_{\textrm{dip}} = \hbar \omega _{a}\hat{\sigma }^{+}\hat{\sigma }^{-}$ is the dipole Hamiltonian with $\omega _{a}$ the resonant frequency of the dipole. We also incorporate the interaction between the TI-QD system and the external field through the Hamiltonian^40^

9 $$\begin{aligned} \hat{H}_{\textrm{exc}} = i\hbar \,\sqrt{k}\, (\hat{a}^{\dag }\varepsilon _{d}\,e^{-i\omega t} - \hat{a}\,\varepsilon _{d}^{*} e^{i\omega t}) + i \hbar \, (\hat{\sigma }^{+}\,\Omega _{R}\,e^{-i\omega t} - \hat{\sigma }^{-}\,\Omega _{R}^{*}\,e^{i\omega t}), \end{aligned}$$

with *k* the total decay rate of the TI due to Ohmic losses to the environment and to its scattering into free space, $\varepsilon _{d}$ the amplitude of the electric field induced on the TIs surface driven by the external field, and $\Omega _{R}= i E_{0}\,d/2\hbar$ the Rabi frequency. In order to eliminate the terms that oscillate too fast in Eq. (9), we apply an unitary transformation based on the well known Baker–Campbell–Hausdorff formula^43^, then we obtain the total Hamiltonian of the system

10 $$\begin{aligned} \hat{H} _{{\textrm{S}}}{} & {} = \hat{H} _{{\textrm{TI}}} + \hat{H} _{{\textrm{dip}}} + \hat{H}_{{\textrm{int}}} + \hat{H}_{{\textrm{exc}}} = \hbar \Delta _{\textrm{TI}}\hat{a}^{\dag }\hat{a} +\hbar \Delta _{\textrm{dip}}\hat{\sigma }^{+}\hat{\sigma }^{-} +\hbar g(r)(\hat{\sigma }^{+}\hat{a}+\hat{a}^{\dag }\hat{\sigma }^{-})\nonumber \\{} & {} \quad + i\hbar (\sqrt{k}\varepsilon _{d} \hat{a}^{\dag }-\sqrt{k}\varepsilon _{d}^{*}\hat{a} +\Omega _{R}\hat{\sigma }^{+}-\Omega _{R}^{*}\hat{\sigma }^{-}), \end{aligned}$$

where $\Delta _{\textrm{TI}} = \Omega -\omega$ and $\Delta _{\textrm{dip}} = \omega _{a} - \omega$ are the detunings between the TI and dipole with the external field.

### Density matrix formalism: from Lindblad’s master equation to entanglement

Now we deal with energy losses from the hybrid to the environment. In the theory of open quantum systems^44^ in which a subsystem interacts with the environment, irreversible evolution due to e.g., the spontaneous emission from an atomic excited level, or due to radiation and dampings, is inevitable. The irreversibility is manifest as this interaction is lost in the environment and cannot be retrieved, which is sometimes referred to as a Markovian (or memory-less) environment. However, such irreversible evolution can still be described by a master equation that generalises the Liouville equation for the density matrix operator. This equation is known as Lindblad’s master equation^45,46^, which has been applied in the study of systems consisting of metallic nanoparticles, quantum dots, and microcavities^16,40,47^. On the other hand, formulating the dynamic evolution in terms of the density matrix will prove useful for studying entanglement. This formalism applies well for our concerns: the hybrid not only pours the TI’s Ohmic losses in the environment/reservoir exciting phonon reservoir modes, the QD also emits dipole radiation that is lost from the hybrid to the environment exciting radiative reservoir modes and the reservoir modes also interact with the EM field operator of the TI and with the QD-dipole operator. Thus, we will satisfactorily describe the dynamics of our open quantum system, duly accounting for losses and damping effects; also we use then such formalism to quantify the entanglement in our system. In our case, Lindblad’s master equations takes the form

11 $$\begin{aligned} \begin{aligned} \frac{d\hat{\rho }}{dt}&= -\frac{i}{\hbar }[\hat{H}_{S}, \hat{\rho }] + \hat{\mathscr {L}}_{{\textrm{TI}}} + \hat{\mathscr {L}}_{{\textrm{QD}}},\\ \hat{\mathscr {L}}_{{\textrm{TI}}}&= -\frac{k}{2} (\hat{a}^{\dag }\hat{a}\hat{\rho } + \hat{\rho } \hat{a}^{\dag }\hat{a} - 2\hat{a}\hat{\rho }\hat{a}^{\dag }),\\ \hat{\mathscr {L}}_{{\textrm{QD}}}&= -\frac{\gamma _{s}}{2} (\hat{\sigma }^{+}\hat{\sigma }^{-}\hat{\rho } + \hat{\rho }\hat{\sigma }^{+}\hat{\sigma }^{-} - 2\hat{\sigma }^{-}\hat{\rho }\hat{\sigma }^{+}) + \frac{\gamma _{d}}{2} (\hat{\sigma }^{+}\hat{\sigma }^{-}\hat{\rho } - \hat{\rho }\hat{\sigma }^{+}\hat{\sigma }^{-} + \hat{\sigma }^{+}\hat{\sigma }^{-}\hat{\rho }\hat{\sigma }^{+} \hat{\sigma }^{-}-\hat{\rho }), \end{aligned} \end{aligned}$$

where $\hat{\mathscr {L}}_{{\textrm{TI}}}$ and $\hat{\mathscr {L}}_{{\textrm{QD}}}$ are the Lindblad operators of the TI and QD, with $\gamma _{s}$ and $\gamma _{d}$ the spontaneous emission rate and dipole dephasing rate of the QD, respectively, and *k* was defined as the total decay rate of the TI, which includes the damping parameter due to Ohmic losses $\gamma _{0}$ and the scattering rate into free space $\gamma _{r}$. The dynamics of the system is essentially described by the optical Bloch equations (OBEs), which correspond to equations of motion of the density matrix elements. For it, we label the base ket of the TI-QD system as $|\beta \, ; m \rangle$, where $\beta =G, X$ correspond to the ground (*G*) and excited (*X*) states of the the dipole respectively, and $m=0,1,2,\dots$ correspond to the states of the TI bosonic surface modes.

We quantify the entanglement of the system using the Peres criterion, according to which if the state of a bipartite system is separable, then all the eigenvalues of its partial transpose are positive^48^. In our case, as the non-zero density matrix elements are given by: $\rho _{Xm, Xm},\rho _{Gm+1,Gn+1},\rho _{Gm+1,Xm},$ and $\rho _{Xm,Gm+1}$, the non-zero density matrix elements of the partial transpose with respect to the excitonic subsystem, i.e. the dipole, are

12 $$\begin{aligned} \begin{aligned} \rho _{Gm,Gm}^{T}&= \rho _{Gm,Gm},\,\,\,\,\, \rho _{Gm+1,Gm+1}^{T} = \rho _{Gm+1,Gm+1},\\ \rho _{Xm+1,Gm}^{T}&= \rho _{Gm+1,Xm},\,\,\,\,\, \rho _{Gm,Xm+1}^{T} = \rho _{Xm,Gm+1}. \end{aligned} \end{aligned}$$

The partial transpose of the density matrix in the basis $\{|X;0\rangle, |G;0\rangle,|X;1\rangle,|G;1\rangle, |X;2\rangle \ldots |G;m\rangle,|X;m+1\rangle,|G;m+1\rangle \}$ applies on the diagonal elements which form $1\times 1$ and $2\times 2$ blocks:

13 $$\begin{aligned} \rho ^{T}= \left( \begin{array}{cccccc} \rho _{X0,X0} &{} &{} &{} &{} &{} \\ &{} \ddots {} &{} &{} &{} &{} \\ &{} &{}\rho _{Gm,Gm} &{}\rho _{Xm,Gm+1} &{} &{} \\ &{} &{}\rho _{Gm+1,Xm}&{}\rho _{Xm+1,Xm+1}&{} &{} \\ &{} &{} &{} &{} \ddots {} &{} \\ &{} &{} &{} &{} &{}\rho _{Gm+1,Gm+1} \end{array} \right). \end{aligned}$$

The eigenvalues corresponding to the left upper and right lower blocks of the above matrix, $\rho _{X0,X0}$ and $\rho _{Gm+1,Gm+1}$, are always positive or zero. On the other hand, the eigenvalues corresponding to the $2\times 2$ blocks, which we called *A*, can be determined using the formula $\lambda _{\pm } = \text{ Tr }\{A\}\pm \sqrt{\text{ Tr }\{A\}^{2}-4\,\text{ det }\{A\}}$, providing

14 $$\begin{aligned} \widetilde{\lambda }_{\pm } = \sqrt{(\rho _{Gm,Gm})^{2}+(\rho _{Xm+1,Xm+1})^{2}-2\rho _{Gm,Gm}\rho _{Xm+1,Xm+1}+4|\rho _{Xm,Gm+1}|^{2}}, \end{aligned}$$

with $\widetilde{\lambda }_{\pm } = \lambda _{\pm }-(\rho _{Gm,Gm}+\rho _{Xm+1,Xm+1})$. From Eq. (14) we have a negative eigenvalue and therefore an entangled state. Also, Eq. (14) implies the condition $|\rho _{Xm,Gm+1}|-\sqrt{\rho _{Gm,Gm}\rho _{Xm+1,Xm+1}}>0$. Thus, entanglement can be quantified by the formula

15 $$\begin{aligned} E(\rho ) = \sum _{m=0, 1, 2\ldots } \text{ max }\big \{0,|\rho _{Xm,Gm+1}|-\sqrt{\rho _{Gm,Gm}\rho _{Xm+1,Xm+1}}\big \}. \end{aligned}$$

Hence, truncating the infinite tower of states of the TI bosonic surface modes to $m = 0,1$ (see Fig. 1) and naming the states of the dipole as $G = 0$ and $X = 1$, we get the effective OBEs of the system (Eq. 22). Therefore, in our model, the measure of TI-QD entanglement is given by the maximum value between zero and the absolute value of the density matrix element $\rho _{1001}$, $E(\rho ) = \text{ max }\big \{0,|\rho _{1001}|\big \}$, namely the density matrix between the hybrid states $\langle X;0 | \rho | G; 1 \rangle$. The component $\rho _{1001}$ is determined by solving the OBEs, a system of differential equations that couple it to the other components of the density matrix, with coefficients that depend on the parameters of the system (see “Methods” section). Graphically, from the right panel of Fig. 1, we see that entanglement $E(\rho )$ is intimately connected with the topological properties of the TI as ultimately the coupling strength *g*(*r*) is a direct signal of the TI’s topological EM response. Our model does not hinge on details of numerical solutions for its description. In that respect, the information of the band topology of the TI sphere is extracted from how sensitive this entanglement is as a function of $\tilde{\alpha }$.

Now we apply our results to a realistic TI-QD hybrid, which is carried out by using appropriate values of the parameters in the effective OBEs (Eq. 22). We assume a CdSe QD interacting with a spherical TI nanoparticle made of TlBiSe$_{2}$. For such TI the parameters are found to be $\mu =1$ and $\varepsilon _{1}(0) = 1 + (\omega _{e}/\omega _{R}) \sim 4$^28^. Further, we take an energy of $\hbar \Omega = 2.2$ eV, a damping parameter due to Ohmic losses $\gamma _{0} = 0.2$ eV and a scattering rate into free-space modes $\gamma _{r} = 2.3\times 10^{-4}$ eV, hence the total decay rate of the TI is $k = \gamma _{0} + \gamma _{r} \approx 0.2$ eV. We choose the TI nanoparticle radius to be $R = 4$ nm, which is embedded in a polymer layer of poly(methyl methacrylate) with permittivity $\varepsilon _{2} = 1.5$^29^. We also consider a spherical CdSe QD with a size of 4 nm, a resonance energy $\hbar \omega _{a} = 2.0$ eV, a spontaneous emission rate $\gamma _{s} = 6.5 \times 10^{-8}$ eV and a dipole dephasing rate $\gamma _{d} = 4.9 \times 10^{-6}$ eV. We set a transition dipole moment $d = 6.4\times 10^{-28}$ Cm^49^. The external laser field was chosen to have a wavelength of $\lambda = 2\pi c/\omega = 550$ nm, then the detunings between the TI and QD with such external field are $\Delta _{\textrm{TI}} = 1.87$ eV and $\Delta _{\textrm{dip}} = 1.84$ eV, respectively. Finally, we suppose that the TI driving field amplitude $\varepsilon _{d}$ approaches the rate of one photon per modified lifetime of the QD, i.e. $|\varepsilon _{d}|^{2} = \Gamma$, where $\Gamma = \gamma _{s} + [4g^{2} k/(k^{2}+4(\Omega -\omega _{a})^{2})]$ is defined as the modified spontaneous emission rate^40^.

**Figure 2:**
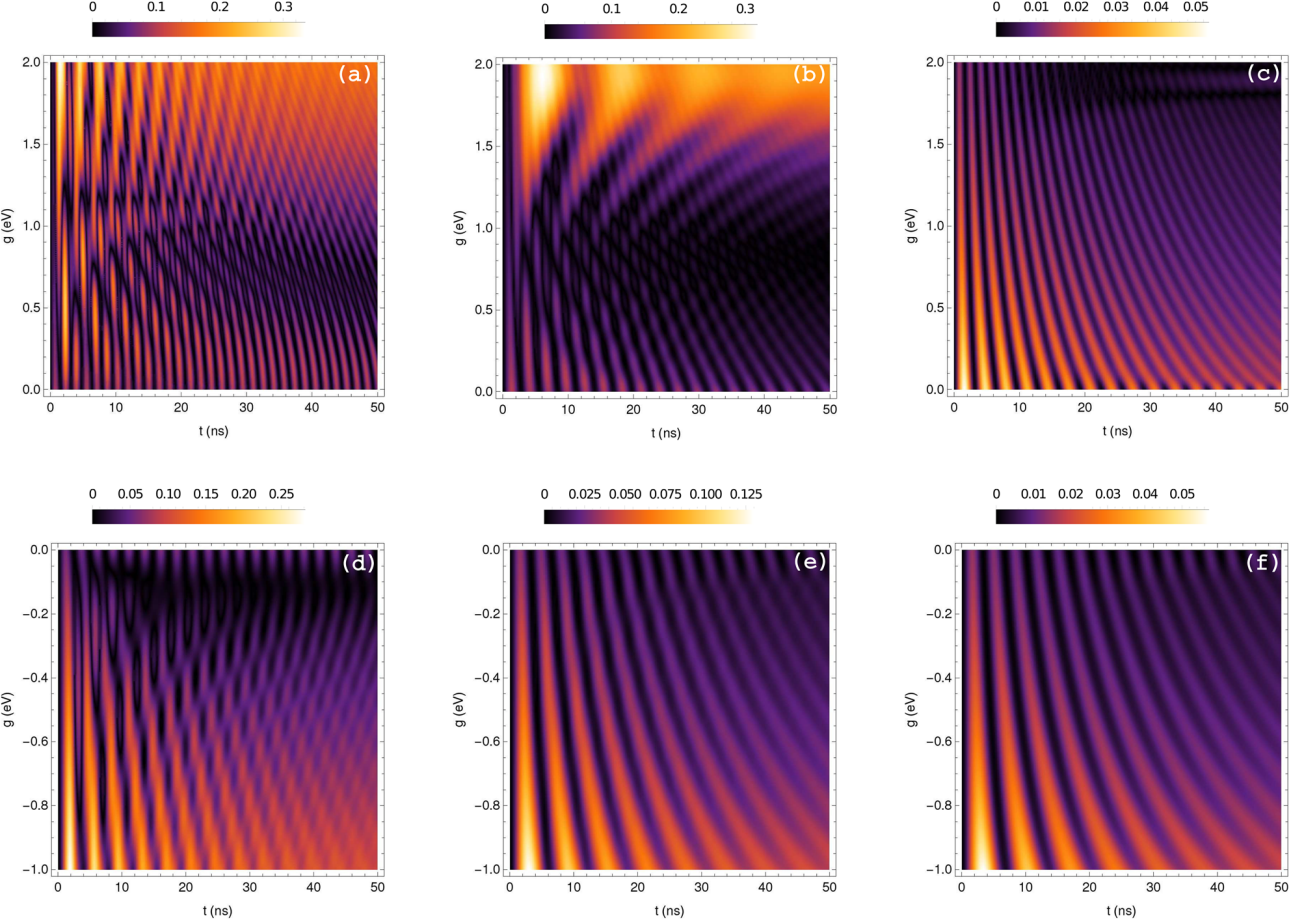
Density plots of entanglement vs coupling strength (g) vs time (t) in LC (up) and TC (bottom) for different values of the parameter $\tilde{\alpha }$: (**a**,**d**) $\tilde{\alpha }= 1/137$, (**b**,**e**) $\tilde{\alpha }= 3/137$, (**c**,**f**) $\tilde{\alpha }= 7/137$.

Figure 2 (up) shows the entanglement of the system as a function of the coupling strength parameter (*g*) and time (*t*) in longitudinal coupling (LC). We choose three different values of $\tilde{\alpha }$: (a) $\tilde{\alpha } = 1/137$, (b) $\tilde{\alpha } = 3/137$, and (c) $\tilde{\alpha } = 7/137$. In Fig. 2a, we observe an entanglement pattern initially with an oscillatory dynamic that tends to be constant, mainly in the interval $g=[1.0-2.0]$ eV. In Fig. 2b, entanglement achieves a maximum value of $E(\rho ) = 0.3$ in the interval $g=[1.2-2.0]$ eV at 4 ns, then decreases gradually until $E(\rho ) = 0.2$ at 50 ns. Figure 2c shows that entanglement has initially a maximum value of $E(\rho )=0.05$ for nearly vanishing coupling strength, which then decreases constantly in time, also we notice that for $g \sim 1.9$ the oscillation of entanglement stops at $\sim$ 18 ns to the faintly resume at $\sim$ 26 ns, which could be considered as a reminiscent of a collapse and revival of entanglement. Finally, it is remarkable that for LC, at $\tilde{\alpha } = [1-3]/137$, above $g \sim 1.5$ entanglement seems more “persistent”, namely exhibiting an almost steady value close to its maximum. Figure 2 (bottom) shows the entanglement of the system as a function of the coupling strength parameter (*g*) and time (*t*) in transverse coupling (TC). Similarly as above, we set the same values for $\tilde{\alpha }$: (d) $\tilde{\alpha } = 1/137$, (e) $\tilde{\alpha } = 3/137$, and (f) $\tilde{\alpha } = 7/137$. We observe that, as a function of $\tilde{\alpha }$, for both couplings LC and TC the maximum value of entanglement decreases monotonically. For TC it is actually absolutely monotonically decreasing in the shown range, while for LC in the $\tilde{\alpha } = [1 - 3]/137$ range it remains constant. Their total decrement is, however, similar of $\sim 1$ order of magnitude with respect to their maximum values for $\tilde{\alpha } = [1 - 7]/137$.

**Figure 3:**
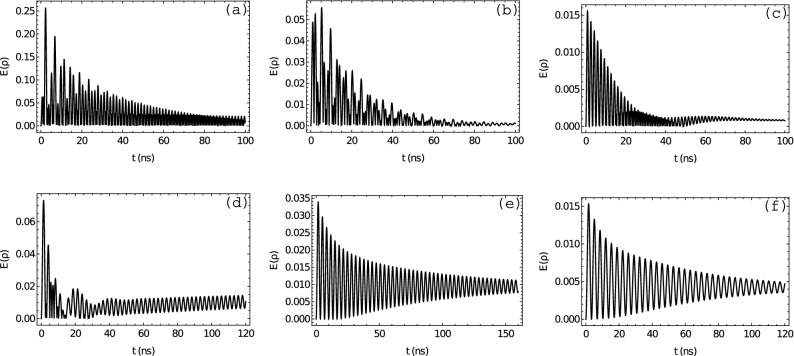
Entanglement $E(\rho )$ vs time in LC (up) and TC (bottom) with (**a**,**d**) $\tilde{\alpha }= 1/137$, (**b**,**e**) $\tilde{\alpha }= 3/137$, and (**c**,**f**) $\tilde{\alpha }= 7/137$. We fix the coupling strength to (**a**) $g = 0.5$ eV, (**b**) $g = 0.8$ eV, (**c**) $g = 1.8$ eV, (**d**) $g = -0.1$ eV, (**e**) $g = -0.05$ eV, and (**f**) $g = -0.05$ eV.

Finally, Fig. 3 shows TI-QD entanglement as a function of time for LC (up) and TC (bottom) with (a, d) $\tilde{\alpha }=1/137$, (b, e) $\tilde{\alpha }=3/137$, and (c, f) $\tilde{\alpha }=7/137$. In other words, Fig. 3 correspond to cuts in the plane $E(\rho )$ vs time of the Density Plots in Fig. 2 for fixed values of the coupling strength *g*: (a) $g = 0.5$ eV, (b) $g = 0.8$ eV, (c) $g = 1.8$ eV, (d) $g = -0.1$ eV, (e) $g = -0.05$ eV, and (f) $g = -0.05$ eV. Here it is remarkable that, despite entanglement has a maximum value higher for LC ($E(\rho )=0.25$) than for TC ($E(\rho ) \approx 0.06$), it decreases to zero for LC; nevertheless for TC, entanglement decreases but achieves a non-zero constant value. We have applied also the above analysis to other TI samples, such as Bi$_2$Te$_3$ and Bi$_2$Se$_3$. For these materials the entanglement pattern exhibits a similar behaviour than for TlBiSe$_{2}$, as depicted in Figs. 2 and 3, showing that our result is not a peculiarity of the particular TI material chosen. A notorious difference is, however, the frequency of the oscillations, which is controlled by the optical properties of the material. The TIs Bi$_2$Te$_3$ and Bi$_2$Se$_3$ have larger static permittivities^50,51^ than TlBiSe$_{2}$, and hence the frequency of the oscillations in the entanglement pattern are shorter.

## Discussion

One of the most intriguing characteristics of 3D TIs is the TME that arises from the nontrivial topology of the underlying band structure. These magnetoelectric phenomena are described by a topological field theory akin to *axion* electrodynamics. For TIs, the *axion* coupling, or $\theta$ term, calculated from the Bloch-state wave function of the system, is quantized in units of the fine-structure constant $\alpha$. Many TME effects have been predicted in TIs, for example: additional Faraday and Kerr rotations due to the TME parameter $\theta$ have been observed in THz spectroscopy of 3D TIs and even a universal TME effect for strained HgTe^39,52–54^, however, these effects are of order $\theta$. Being $\theta$ proportional to $\alpha$, the typical (non-topological) optical responses usually overwhelm topological ones. A good possibility to test the topological non-triviality in the EM response is by making its strength comparable to the baseline EM signals. This was one of our main motivations and to do so, we couple a TI sample with a quantum system, thus the typical and topological responses result both of $\sim \mathscr {O}(\alpha )$. Specifically, we found that entanglement in a realistic TI-QD hybrid consisting of a TI nanosphere made of TlBiSe$_{2}$ interacting with a CdSe QD, embedded in a polymer layer of poly(methyl methacrylate) subject to an impulse magnetic field, exhibits strong direct signatures of the TME effect. Performing experiments to measure signals of either the TME effect or entanglement certainly are very challenging tasks. Subtle technical details that could potentially hamper these minute signals will need to be addressed. These would be highly dependent on a given experimental setup. At the moment, these are hard to foresee and we prefer to focus on its theoretical prediction, characterization and its potential. We analyze and interpret these signatures focusing on the dependence of entanglement upon: (a) the TME parameter, (b) spatial configurations variables and (c) temporal evolution.

Regarding the dependence of entanglement on the TME parameter, from Fig. 2 we observe that, for LC and TC, the maximum value of entanglement tends to decrease as $\tilde{\alpha }$ increases. This is somewhat paradoxical and deserves a more thorough understanding that we decide to defer. Nevertheless, this can be taken advantage of if one is concerned with the manipulation, control and characterization of entangled states in TI-QD hybrids. Another feature is that for both LC and TC, at lowest $\tilde{\alpha }$ the oscillatory patterns in the density plots (Fig. 2) present some distortion. The breaking of these patterns suggests an intriguing kind of interference, which, however, requires a finer analysis that lies beyond our scope.

With respect to the spatial dependence, firstly, we can confirm that the entanglement decreases with the increasing TI-QD distance (not shown for lack of space), which is as expected and consistent^28,29^. Also, the direction of the electric field of the TI at the position of the QD relative to its dipole operator, what we term longitudinal *L*, or transverse *T* “polarizations” $\mathfrak {p}$ of the coupling, influences entanglement too. In fact, as function of *g* and $\tilde{\alpha }$ (Fig. 2) and also for fixed *g* and $\tilde{\alpha }$ as function of *t* (Fig. 3), entanglement show notorious differences for different $\mathfrak {p}$, which are more revealing by analyzing the time dependence: the time dependence of entanglement is ultimately determined by the time evolution of the density matrix elements. Though a comprehensive understanding is in order, an oscillatory behaviour is not unexpected. For both $\mathfrak {p}$ and most ranges of $\tilde{\alpha }$ and *g*, entanglement oscillates with some sort of attenuation with a generic time profile $\sim \mathscr {E}^{\tilde{\alpha }}_{\mathfrak {p}} (g, t) \, e^{\pm \, i\, \omega ^{\tilde{\alpha }}_{\mathfrak {p}}(g) t}$, with $\mathscr {E}^{\tilde{\alpha }}_{\mathfrak {p}} (g, t)$ an unspecified attenuating function. For a given *g* and the same $\tilde{\alpha }$, the angular frequency for LC is nearly twice as large as for TC (Figs. 2, 3), that is $\omega _{LC} \,\,\dot{\approx }\,\, 2 \, \omega _{TC}$, the over-dot meaning “for the same $\tilde{\alpha }$ and for a fixed *g*”. As *g* are interaction energies, omitting the label $\tilde{\alpha }$ and the *g* dependence, the oscillatory behaviours are governed by $e^{\pm i \omega _{\mathfrak {p}} \,t} = e^{\pm i \mathfrak {E}_{\mathfrak {p} } \,t/\hbar }$, where $\mathfrak {E}_{\mathfrak {p}} = \hbar \omega _{\mathfrak {p}}$ corresponds to an energy scale of the interaction coupling, which, for fixed $\tilde{\alpha }$ and for a given value of *g*, is $\mathfrak {p}$ dependent. Namely, the energy scale of the interaction sets the time scale for the oscillation of entanglement, thus Eq. (8) explains the factor of $\sim$2.

Regarding the attenuating functions, though in Fig. 2 there are regions in the parameter space which may suggest no time-oscillating entanglement at all, this is due to very rapid oscillations, which, for $\mathfrak {p}=$TC attenuates to a constant non-zero value, while for $\mathfrak {p}=$LC it does to zero. We do not attempt a thorough analysis of these attenuating functions, yet we can provide some insight. First, this is not specific to the values of $\tilde{\alpha }$ nor the ranges of *g* for each $\mathfrak {p}$. In all the density plots (Fig. 2), entanglement for a fixed $\tilde{\alpha }$ and different $\mathfrak {p}$, focusing on the range of *g* for which the entanglement seem to vanish, we consistently observe that for LC it tends to zero, while for TC it does not (Fig. 3). This feature can be exploited in the following ways. If in a given experimental setup the TI-QD coupling were “unpolarized” but for some reason one can observe whether the states are entangled or not, then this observation can serve to discriminate between different $\mathfrak {p}$, if this was otherwise impossible to asses. In quantum-information or -computing devices, decoherence poses serious problems, one of the main sources for it being the presence of entanglement. Thus, if in the future a setup like ours were found to be appropriate for realizations in these contexts, then we envision another interesting application, namely the a priori choice of $\mathfrak {p}$ = TC and the TMEP could foster fault-tolerant quantum computation by masking the effects due to decoherence. As to the different time-behaviour of the attenuating functions, we can hypothesize that it might be a consequence of either: (a) the damping effects due to Ohmic losses being larger the stronger the coupling as the induced excitations on the TI’s surface dissipate faster for LC. This is untenable though, as it implies, contrary to what we have found, that we merely have to wait longer for TC entanglement to decay to zero; (b) a phenomenon related to entanglement sudden death which has been observed occasionally when there is evolution in a dissipative environment^55^; or (c) given that for TC the states of the system always keeps probing the TMEP effects of the TI, while for LC it does not, hinging on the fact that the observed entanglement is entirely due to the TMEP, a more speculative hypothesis would be that the quantum mechanical evolution of the interaction between the TI-QD immersed in the external field could trigger a quantum phase transition not necessarily related to one in the band structure of the TI, but rather to the rearrangements of the patterns of entanglement in the full interacting system. The perspective that topological order should be understood from an entanglement point of view^56^ endorses this hypothesis and therefore we believe it deserves to be studied further and, if it were possible, subsequently subject to experimental scrutiny.

We found that the topological entanglement between a TI and QD immersed in an external driving magnetic field depends on the configuration of the system, e.g.; TI-QD distance (relative to the size of the TI); the direction of the EM field, at the position of the QD, relative to its dipole operator i.e., on whether the coupling if transverse or longitudinal. The reported phenomena are also sensitive to the value of the topological magnetoelectric parameter TMEP of the TI. Furthermore we found an interesting time dependence. We would like to point out that to the best of our knowledge, it is the first time that these kinds of results are reported. Several explanations, interpretations and hypothesis of our results have been provided and wide range of possible applications in *topological quantum information and computation*, such term was introduced to name quantum computation using topological insulators^2^ are envisioned. It may well be that our findings result of relevance for the study and characterization of quantum devices interacting with topological materials, or for conceiving quantum information sensors where the aforementioned variables can be used to witness quantum entanglement or characteristic features of the interaction. The theoretical model developed in this work can be extended to other topological materials, e.g., topological quantum wires, topological Weyl semimetals and Dirac semimetals, problems that can be further studied within the same scheme.

## Methods

### Optical Bloch equations

In this section, we present the deduction of the optical Bloch equations (OBEs) of the TI-QD hybrid modeled as an open quantum system: we compute the $\langle \alpha ;n|(\,\,)|\beta ; m\rangle$ density matrix elements of Lindblad’s master equation (Eq. 11),

16 $$\begin{aligned} \langle \alpha n|\dot{\hat{\rho }}|\beta m\rangle = -\frac{i}{\hbar } \big ( \underbrace{\langle \alpha n|\hat{H}_{S}\hat{\rho }|\beta m\rangle }_{(L)} - \underbrace{\langle \alpha n|\hat{\rho } \hat{H}_{S}|\beta m\rangle }_{(M)} \big ) + \underbrace{\langle \alpha n|\hat{\mathscr {L}}_{{\textrm{TI}}}\cdot \hat{\rho }| \beta m\rangle }_{(N)} +\underbrace{\langle \alpha n|\hat{\mathscr {L}}_{{\textrm{QD}}}\cdot \hat{\rho }| \beta m\rangle }_{(O)}, \end{aligned}$$

where, for simplicity, we have written $|\beta ; m \rangle = |\beta m \rangle$. We replace the total Hamiltonian (Eq. 10) in the above expression and solve the term *L*,

17 $$\begin{aligned} \begin{aligned} \langle \alpha n|\hat{H}_{S}\hat{\rho }|\beta m\rangle&= \langle \alpha n|\big [ \hbar \Delta _{{\textrm{TI}}}\hat{a}^{\dag }\hat{a} +\hbar \Delta _{{\textrm{QD}}}\hat{\sigma }^{+}\hat{\sigma }^{-} +\hbar g(r) (\hat{\sigma }^{+}\hat{a}+\hat{a}^{\dag }\hat{\sigma }^{-}) + i\hbar (\sqrt{k}\,\varepsilon _{d}\,a^{\dag }\\&\quad -\sqrt{k}\,\varepsilon _{d}^{*}\,\hat{a} +\Omega _{R}\hat{\sigma }^{+}-\Omega _{R}^{*}\hat{\sigma }^{-}) \big ]\hat{\rho }|\beta m\rangle,\\&= \hbar \Delta _{{\textrm{TI}}}\, n\langle \alpha n|\hat{\rho }|\beta m\rangle +\quad \hbar \Delta _{{\textrm{QD}}} \langle X n|\hat{\rho }|\beta m\rangle \delta _{\alpha X}\\&\quad + \hbar g(r) \sqrt{n+1}\langle G n+1|\hat{\rho }|\beta m\rangle \delta _{\alpha X}+\hbar g(r)\sqrt{n}\langle X n-1|\hat{\rho }|\beta m\rangle \delta _{\alpha G}\\&\quad + i\hbar \big (\sqrt{k}\, \varepsilon _{d}\sqrt{n}\langle \alpha n-1|\hat{\rho }|\beta m\rangle -\sqrt{k}\,\varepsilon _{d}^{*}\sqrt{n+1}\langle \alpha n+1|\hat{\rho }|\beta m\rangle \\&\quad + \Omega _{R}\langle G n|\hat{\rho }|\beta m\rangle \delta _{\alpha X}-\Omega _{R}^{*}\langle X n|\hat{\rho }|\beta m\rangle \delta _{\alpha G}\big ), \end{aligned} \end{aligned}$$

with $\delta _{ij}$ a Kronecker delta. Similarly, we solve the term *M*

18 $$\begin{aligned} \begin{aligned} \langle \alpha n|\hat{\rho }\hat{H}_{S}|\beta m\rangle&= \langle \alpha n|\hat{\rho }\big [ \hbar \Delta _{{\textrm{TI}}}\hat{a}^{\dag }\hat{a} +\hbar \Delta _{{\textrm{QD}}}\hat{\sigma }^{+}\hat{\sigma }^{-} +\hbar g(r) (\hat{\sigma }^{+}\hat{a}+\hat{a}^{\dag }\hat{\sigma }^{-}) + i\hbar (\sqrt{k}\,\varepsilon _{d}\,a^{\dag }\\&\quad -\sqrt{k}\,\varepsilon _{d}^{*}\,\hat{a} +\Omega _{R}\hat{\sigma }^{+}-\Omega _{R}^{*}\hat{\sigma }^{-}) \big ]|\beta m\rangle,\\&= \hbar \Delta _{{\textrm{TI}}}\, m\langle \alpha n|\hat{\rho }|\beta m\rangle + \hbar \Delta _{{\textrm{QD}}} \langle \alpha n|\hat{\rho }|X m\rangle \delta _{\beta X}\\&\quad + \hbar g(r) \sqrt{m}\langle \alpha n|\hat{\rho }|X m-1\rangle \delta _{\beta G}+\hbar g(r)\sqrt{m+1}\langle \alpha n|\hat{\rho }|G\,m+1\rangle \delta _{\beta X}\\&\quad + i\hbar \big (\sqrt{k}\, \varepsilon _{d}\sqrt{m+1}\langle \alpha n|\hat{\rho }|\beta m+1\rangle -\sqrt{k}\,\varepsilon _{d}^{*}\sqrt{m}\langle \alpha n|\hat{\rho }|\beta m-1\rangle \\&\quad + \Omega _{R}\langle \alpha n|\hat{\rho }|X m\rangle \delta _{\beta G} -\Omega _{R}^{*}\langle \alpha n|\hat{\rho }|G m\rangle \delta _{\beta X}\big ). \end{aligned} \end{aligned}$$

Solving (*N*) and (*O*),

19 $$\begin{aligned} \langle \alpha n|\hat{\mathscr {L}}_{{\textrm{TI}}}|\beta m\rangle{} & {} = \langle \alpha n|-\frac{k}{2}(\hat{a}^{\dag }\hat{a}\hat{\rho }+\hat{\rho } \hat{a}^{\dag }\hat{a}-2\hat{a}\hat{\rho }\hat{a}^{\dag })|\beta m\rangle,\nonumber \\{} & {} = -\frac{k}{2}\big [n\langle \alpha n|\hat{\rho }|\beta m\rangle +m\langle \alpha n|\hat{\rho }|\beta m\rangle -2\sqrt{n+1}\sqrt{m+1}\langle \alpha n+1|\hat{\rho }|\beta m+1\rangle \big ]. \end{aligned}$$

20 $$\begin{aligned} \langle \alpha n|\hat{\mathscr {L}}_{{\textrm{QD}}}|\beta m\rangle{} & {} = \langle \alpha n| -\frac{\gamma _{s}}{2}(\hat{\sigma }^{+}\hat{\sigma }^{-}\hat{\rho }+\hat{\rho }\hat{\sigma }^{+}\hat{\sigma }^{-}-2\hat{\sigma }^{-}\hat{\rho }\hat{\sigma }^{+})\nonumber \\{} & {} \quad + \frac{\gamma _{d}}{2}(\hat{\sigma }^{+}\hat{\sigma }^{-}\hat{\rho }-\hat{\rho }\hat{\sigma }^{+}\hat{\sigma }^{-}+\hat{\sigma }^{+}\hat{\sigma }^{-}\hat{\rho }\hat{\sigma }^{+}\hat{\sigma }^{-}-\hat{\rho })|\beta m\rangle,\nonumber \\{} & {} = -\frac{\gamma _{s}}{2}\big [\langle X n|\hat{\rho }|\beta m\rangle \delta _{\alpha X}+\langle \alpha n|\hat{\rho }|X m\rangle \delta _{\beta X}-2\langle X n|\hat{\rho }|X m\rangle \delta _{\alpha G}\delta _{\beta G}\big ]\nonumber \\{} & {} \quad + \frac{\gamma _{d}}{2}\big [\langle X n|\hat{\rho }|\beta m\rangle \delta _{\alpha X}-\langle \alpha n|\hat{\rho }|X m\rangle \delta _{\beta X}+\langle X n|\hat{\rho }|X m\rangle \delta _{\alpha X}\delta _{\beta X}-\langle \alpha n|\hat{\rho }|\beta m\rangle \big ]. \end{aligned}$$

Therefore, substituting the previous results into Eq. (17) we have

21 $$\begin{aligned} \begin{aligned} \dot{\rho }_{\alpha n\beta m}&= -\frac{i}{\hbar }\big (\hbar \Delta _{{\textrm{TI}}} n\rho _{\alpha n\beta m} +\hbar \Delta _{{\textrm{QD}}}\rho _{X n\beta m}\delta _{\alpha X} +\hbar g(r)\sqrt{n+1}\rho _{G n+1\beta m}\delta _{\alpha X} +\hbar g(r)\sqrt{n}\rho _{X n-1\beta m}\delta _{\alpha G}\\&\quad + i\hbar \sqrt{k}\,\varepsilon _{d}\sqrt{n}\rho _{\alpha n-1\beta m}-i\hbar \sqrt{k}\,\varepsilon _{d}^{*}\sqrt{n+1}\rho _{\alpha n+1\beta m} +i\hbar \Omega _{R}\rho _{G n\beta m}\delta _{\alpha X}-i\hbar \Omega _{R}^{*}\rho _{X n\beta m}\delta _{\alpha G}\big )\\&\quad + \frac{i}{\hbar }\big (\hbar \Delta _{{\textrm{TI}}} m\rho _{\alpha n\beta m}+\hbar \Delta _{{\textrm{QD}}} \rho _{\alpha n X m}\delta _{\beta X} +\hbar g(r)\sqrt{m}\rho _{\alpha n X m-1}\delta _{\beta G} +\hbar g(r)\sqrt{m+1}\rho _{\alpha n G m+1}\delta _{\beta X}\\&\quad +i\hbar \sqrt{k}\,\varepsilon _{d}\sqrt{m+1}\rho _{\alpha n\beta m+1}-i\hbar \sqrt{k}\,\varepsilon _{d}^{*}\sqrt{m}\,\rho _{\alpha n\beta m-1} +i\hbar \,\Omega _{R}\,\rho _{\alpha n X m}\delta _{\beta G}-i\hbar \,\Omega _{R}^{*}\,\rho _{\alpha n G m}\delta _{\beta X}\big )\\&\quad - \frac{k}{2}\big (n\rho _{\alpha n\beta m}+m\rho _{\alpha n\beta m}-2\sqrt{(n+1)}\sqrt{(m+1)}\rho _{\alpha n+1\beta m+1}\big )\\&\quad - \frac{\gamma _{s}}{2}\big (\rho _{X n\beta m}\delta _{\alpha X}+\rho _{\alpha n X m}\delta _{\beta X}-2\rho _{X n X m}\delta _{\alpha G}\delta _{\beta G}\big )\\&\quad + \frac{\gamma _{d}}{2}\big (\rho _{X n\beta m}\delta _{\alpha X}-\rho _{\alpha n X m}\delta _{\beta X}+\rho _{X n X m}\delta _{\alpha X}\delta _{\beta X}-\rho _{\alpha n\beta m}\big ), \end{aligned} \end{aligned}$$

where $\alpha,\beta =G, X$ correspond to the ground (*G*) and excited (*X*) states of the the dipole, and $n, m=0,1,2,\dots$ correspond to the states of the TI bosonic surface modes. Putting $G,X = 0,1$ and truncating the tower of states to $n,m = 0,1$, we obtain the effective OBEs of the system:

22 $$\begin{aligned} \begin{aligned} \dot{\rho }_{0000}&= -\frac{1}{2}\gamma _{d}\rho _{0000}+k\rho _{0101}+\gamma _{s} \rho _{1010} + \sqrt{k}(\varepsilon _{d}\rho _{0001}+\varepsilon _{d}^{*}\rho _{0100}) + \Omega _{R}\rho _{0010} +\Omega _{R}^{*}\rho _{1000},\\ \dot{\rho }_{0001}&= -\frac{1}{2}\rho _{0001}(k+\gamma _{d})+\Omega _{R}^{*}\rho _{1001} - \sqrt{k}\varepsilon _{d}^{*}\rho _{0000}-i(\Delta _{{\textrm{TI}}}\rho _{0001}+g\rho _{0010}),\\ \dot{\rho }_{0010}&= -\rho _{0010}\bigg (\frac{\gamma _{s}}{2}+\gamma _{d}\bigg ) +\sqrt{k}\varepsilon _{d}^{*}\rho _{0110} -i(g\rho _{0001}+\Delta _{{\textrm{QD}}}\rho _{0010}) -\Omega _{R}^{*}(\rho _{0000}-\rho _{1010}),\\ \dot{\rho }_{0100}&= -\frac{1}{2}\rho _{0100}(k+\gamma _{d})+\Omega _{R}\rho _{0110} -\sqrt{k}\varepsilon _{d}\rho _{0000}+i(\Delta _{{\textrm{TI}}}\rho _{0100}+g\rho _{1000}),\\ \dot{\rho }_{0101}&= -k\rho _{0101}-\sqrt{k}(\varepsilon _{d}^{*}\rho _{0100}+\varepsilon _{d}\rho _{0001}) -ig(\rho _{0110}-\rho _{1001}),\\ \dot{\rho }_{0110}&= -\rho _{0110}\bigg (\frac{k+\gamma _{s}}{2}+\gamma _{d} -i\Delta _{{\textrm{TI}}} +i\Delta _{{\textrm{QD}}}\bigg ) -\Omega _{R}^{*}\rho _{0100}-\sqrt{k}\varepsilon _{d}\rho _{0010} -ig(\rho _{0101}-\rho _{1010}),\\ \dot{\rho 
}_{1000}&= -\rho _{1000}\bigg (\frac{\gamma _{s}}{2}+\gamma _{d}\bigg ) +\sqrt{k}\varepsilon _{d}\rho _{1001} +i(g\rho _{0100}+\Delta _{{\textrm{QD}}}\rho _{1000}) +\Omega _{R}(\rho _{1010}-\rho _{0000}),\\ \dot{\rho }_{1001}&= -\rho _{1001} \bigg (\frac{k+\gamma _{s}}{2}+\gamma _{d} +i\Delta _{{\textrm{TI}}} -i\Delta _{{\textrm{QD}}}\bigg ) -\Omega _{R}\rho _{0001}-\sqrt{k}\varepsilon _{d}^{*}\rho _{1000} +ig(\rho _{0101}-\rho _{1010}),\\ \dot{\rho }_{1010}&= -\rho _{1010}(\gamma _{s}+\gamma _{d}) -\Omega _{R}\rho _{0010} -\Omega _{R}^{*}\rho _{1000} +ig (\rho _{0110}-\rho _{1001}). \end{aligned} \end{aligned}$$

## Data Availability

All data generated or analysed during this study are included in this published article.
